# A new treatment for canine B-cell lymphoma based on a recombinant single-domain antibody immunotoxin derived from *Pseudomonas aeruginosa* exotoxin A

**DOI:** 10.3389/fvets.2025.1491934

**Published:** 2025-04-04

**Authors:** Ana S. André, Joana N. R. Dias, Isa Moutinho, Joana Loureiro, Ana Leonardo, Sara Nogueira, Rafaela P. Marimon, Pedro Bule, Jorge Correia, Rui Malhó, Lurdes Gano, João D. G. Correia, Solange Gil, João Gonçalves, Ira Pastan, Luís Tavares, Frederico Aires-da-Silva

**Affiliations:** ^1^Center of Interdisciplinary Research in Animal Health (CIISA), Faculty of Veterinary Medicine, University of Lisbon, Lisbon, Portugal; ^2^Associate Laboratory for Animal and Veterinary Sciences (AL4AnimalS), Lisbon, Portugal; ^3^Biosystems and Integrative Sciences Institute, Faculdade de Ciências, Universidade de Lisboa, Lisbon, Portugal; ^4^Centro de Ciências e Tecnologias Nucleares and Departamento de Engenharia e Ciências Nucleares, IST, Universidade de Lisboa, Lisbon, Portugal; ^5^Research Institute for Medicines (iMed.ULisboa), Faculdade de Farmácia, Universidade de Lisboa, Lisbon, Portugal; ^6^Laboratory of Molecular Biology, Center for Cancer Research, National Cancer Institute, National Institutes of Health, Bethesda, MD, United States

**Keywords:** canine B-cell lymphoma, immunotoxin therapy, recombinant single-domain antibody, comparative oncology, targeted cancer therapeutic

## Abstract

Canine lymphoma is one of the most common and aggressive hematopoietic tumors in dogs. Despite recent advances in veterinary cancer treatments, the lack of specificity, side effects, and resistance to conventional chemotherapies has opened an urgent need to develop more targeted and safe therapeutics to address this unmet need in dogs. Thus, in the present study, we aimed to generate a new class of therapeutics based on a recombinant single-domain antibody (sdAb) immunotoxin derived from the PE38 *Pseudomonas aeruginosa* exotoxin A. For this purpose, we fused the PE38 toxin with the specific C5 sdAb antibody, previously developed by our group for canine B-cell lymphoma. This resulted in a stable and highly specific C5-PE38 immunotoxin against canine B-cell lymphoma. The C5-PE38 immunotoxin revealed a potent cytotoxic activity (EC50 = 9.50 ± 0.04 μg/mL) against CLBL-1 canine B-cell lymphoma cells, while promoting inhibition of protein synthesis and, consequently, cell death. Importantly, *in vivo* results in a CLBL-1 xenograft mouse model demonstrated specific targeted tumor uptake and strong tumor growth inhibition in C5-PE38 treated mice compared with control vehicle-treated mice. The results obtained provide new data validating immunotoxins and recombinant sdAb-PE38 based scaffolds as a novel and promising anti-cancer therapy for the treatment of dog-related tumors, while contributing to comparative oncology.

## 1 Introduction

Over the last few years, remarkable advances have been made in the field of oncology, leading to enhanced therapeutic options for cancer patients. Nevertheless, cancer remains a leading cause of mortality, affecting not only humans but also companion animals, particularly dogs. Non-Hodgkin lymphoma (NHL) is one of the most common hematological neoplasias in both species (1, 2). In humans, NHL accounts for 90% of all lymphomas and 85–90% of B-cell cases. This neoplasia usually develops in the lymph nodes, and the most aggressive forms are diffuse large B-cell lymphoma (DLBCL) and Burkitt’s lymphoma (BL) (3, 4). In dogs, canine lymphoma is one of the most common cancers, accounting for approximately 7–24% of all canine neoplasias and 83% of all canine hematopoietic tumors. Canine lymphoma can present several subtypes; however, the multicentric form is the most common and is usually diagnosed as intermediate-to-high-grade lymphoma of B-cell origin (5, 6). Owing to the shared molecular, genetic, and histopathologic features of canine lymphoma, dogs have been suggested as a valuable animal model for the development of novel therapeutics for human NHL (5). In canine lymphoma treatment, conventional chemotherapy often achieves temporary remission but is rarely curative, with most patients experiencing relapse with lethal, drug-resistant lymphoma (6). This lack of specificity, along with the side effects and drug resistance associated with conventional chemotherapies, underscores the urgent need for more specific and safer therapeutics to address this unmet need in dogs.

In the pursuit of more precise and potent cancer treatments, the strategy of exploring recombinant antibodies conjugated with cytotoxic agents, such as toxins, has garnered significant attention (7). This innovative approach, based on this emerging class of therapeutics named immunotoxins, offers distinct advantages over conventional chemotherapy methods by specifically targeting tumor cells and delivering the toxin molecule into the cells. By employing antibodies that selectively bind to tumor-specific antigens, this approach overcomes the lack of specificity associated with traditional chemotherapies. Furthermore, it harnesses toxins, a unique class of molecules, to combat chemotherapy resistance effectively. Toxin domains used in immunotoxins are derived from bacterial sources, such as *Pseudomonas aeruginosa* exotoxin A or diphtheria toxin, as well as plant toxins, such as ricin and saporin (8). These toxins exhibit potent cytotoxicity and the ability to induce cell death with remarkable efficiency, underscoring their therapeutic potential as promising alternatives to conventional small molecule-based drugs (7–10). While immunotoxins hold significant promise for targeted cancer therapies, challenges such as immunogenicity and the development of anti-drug antibodies (ADAs) must be addressed to ensure sustained efficacy. Advancements in protein engineering, such as PEGylation and targeted mutagenesis to reduce immunogenic epitopes, underscore the potential of immunotoxins as an innovative and selective approach for cancer treatment (7, 11).

Currently, there are three approved immunotoxins for clinical use in human applications. Denileukin diftitox (Ontak®) was first approved by the US Food and Drug Administration (FDA) in 1999 for the treatment of relapsed or refractory cutaneous human T-cell lymphoma (12). In 2018, Moxetumomab pasudotox (Lumoxiti®) and Tagraxofusp (Elzoris®) were approved for the treatment of relapsed and refractory human hairy cell leukemia and blastic plasmacytoid dendritic cell neoplasm, respectively (13, 14). Ontak® and Elzoris® use a truncated diphtheria toxin, DAB389, fused with Interleukin 2 (IL-2) and Interleukin 3 (IL-3), respectively. Moxetumomab pasudotox consists of an anti-CD22 disulfide-stabilized antibody fragment (dsFv) fused to a truncated form of *Pseudomonas aeruginosa* Exotoxin A (PE), named PE38 (15). PE is one of the most common toxins used in the development of recombinant immunotoxins. The PE toxin is composed of three structural domains: the binding domain (I), the processing domain (II), and the catalytic domain (III). The most common truncated form of PE used in immunotoxins, including Moxetumomab pasudotox, is PE38 (10, 16). The mechanism of action of immunotoxins depends on two key factors: target binding and internalization and inhibition of protein synthesis. Since the receptor-binding domain (I) on the toxin is replaced with a target-specific antibody, the antibody is entirely responsible for the targeting and internalization of the entire immunotoxin. Thus, it is important to select a highly specific and stable antibody scaffold that can be used for conjugation with the toxin domain (17–20).

Over the past years, we have been demonstrating the potential of rabbit-derived single-domain antibodies (sdAbs) for several therapeutic applications (21–26). These fragments represent the smallest portions derived from conventional IgGs responsible for antigen binding. Owing to reduced size (12–15 kDa), they present higher tumor penetration and accessibility to targets that are not easily reached by large-size conventional IgG monoclonal antibodies (150 kDa). Furthermore, they exhibit high stability, reduced immunogenicity, and low manufacturing cost (21–29). Importantly, sdAbs can be easily conjugated to other biological payloads, such as therapeutic proteins or toxins, and used as drug delivery systems. In addition, since sdAbs lack the Fc domain of conventional IgGs, nonspecific uptake in tissues that highly express Fc receptors is low (27, 30). These unique properties make sdAbs excellent antibody scaffolds for the construction of recombinant immunotoxins. Thus, in this study, we aimed to explore a new treatment approach for canine B-cell lymphoma based on the development of a recombinant sdAb immunotoxin, while contributing to comparative oncology. To this end, we fused the truncated PE38 toxin domain derived from *Pseudomonas aeruginosa* exotoxin A with a rabbit-derived sdAb, termed C5, which we recently developed and characterized for canine B-cell lymphoma (23).

## 2 Materials and methods

### 2.1 Cell culture

The canine B-cell lymphoma cell line, CLBL-1, was provided by Dr. Barbara Rutgen (University of Vienna, Austria) (31, 32) and was maintained in RPMI-1640 medium (Gibco, Thermo Fisher Scientific, United States) supplemented with 10% FBS (Gibco, Thermo Fisher Scientific, United States) and penicillin 100 U/mL/ streptomycin 0.1 mg/mL (Gibco, Thermo Fisher Scientific, United States) in a humidified atmosphere at 37° C with 5% CO_2_. Peripheral blood mononuclear cells (PBMCs) were isolated from canine blood using Ficoll-Paque™ PLUS (Cytiva), according to the manufacturer’s protocol. The PBMCs were maintained in RPMI-1640 medium, GlutaMAX™, supplemented with 10% FBS, penicillin 100 U/mL/streptomycin 0.1 mg/mL, 1% Sodium Pyruvate, 1% MEM Non-Essential Amino Acids Solution and 0.1% HEPES (Gibco, Thermo Fisher Scientific, United States) in a humidified atmosphere at 37° C with 5% CO_2_.

### 2.2 Structure prediction, construction and expression of C5-PE38

The tridimensional structure of the recombinant C5-PE38 immunotoxin was predicted using the Rosettafold deep learning-based modeling method on the Baker lab’s Robetta server (33). The best model obtained presented a confidence level of 0.77. Visual representations of the structure were produced using the UCSF ChimeraX software (34). C5-PE38 is composed by the sdAb C5 clone (23) fused with a peptide linker (SSGGGGSGGGGGGSSRSS) to PE38, the truncated portion of *Pseudomonas aeruginosa* exotoxin A. To construct the C5-PE38 immunotoxin, C5 and PE38 DNA fragments were amplified individually, using specific primers, from the pET21a-C5 (23) and pRB391 (Dr Ira Pastan lab) vectors, respectively. Then, C5 and PE38 were fused using a PCR overlap extension. The resulting C5-PE38 PCR product was gel-purified, digested with *NheI* and *XhoI* restriction enzymes and cloned into pET-21a expression plasmid (Sigma-Aldrich, St. Louis, MO, United States). A His-tag and HA-tag were added to the C-terminal region of the construct to allow purification through nickel affinity columns and detection in Western blot (WB), ELISA, and immunofluorescence assays. The C5-PE38 construct was confirmed by standard DNA Sanger sequencing methods using Eurofins. The expression, purification and refolding of the C5-PE38 was performed as previously described (23). Briefly, following expression, bacteria were collected by centrifugation (1,500 × g, 15 min, 4°C), and resuspended in 50 mL of initial buffer (50 mM HEPES, 1 M NaCl, 10 mM Imidazole, 6 M Urea, 5 mM CaCl2, 1 mM *β*-mercaptoethanol, and pH = 8) supplemented with protease inhibitors (Sigma, Merck, Germany). Cells were lysed by sonication. and inclusion bodies were harvested by centrifugation (7,500 × g, 30 min, 4°C). Refolding was performed by step wise dialysis, reducing the urea concentration from 6 M to 0 M. Finally, C5-PE38 was purified by size exclusion chromatography (SEC). C5-PE38 purity was analyzed by SDS-PAGE gel with 15% acrylamide under denaturing conditions.

### 2.3 Cell ELISA

To evaluate the specific binding activity of C5-PE38 against canine B-cell lymphoma cells, a cell-based ELISA was performed as previously reported (21). Briefly, 60 × 10^3^ CLBL-1 or canine PBMCs cells (control) were blocked with PBS (Gibco, Thermo Fisher Scientific, United States) with 1% BSA (Sigma, Merck, Germany) for 30 min, washed with cold PBS, and incubated with C5-PE38 at different concentrations (0.5–10 μg/mL) for 45 min at 4°C. Cells were then washed with cold PBS, and HRP-conjugated anti-HA-tag secondary antibody (Roche, Switzerland) at 1:3000 in PBS-BSA 1% was added to each well and incubated for 45 min at 4°C. Following incubation and washing with PBS, ABTS substrate solution (Sigma, Merck, Germany) was added, and the optical density (OD) was measured using a microplate reader (Bio-Rad Laboratories, United States) at 405 nm.

### 2.4 C5-PE38 *in vitro* serum stability

To evaluate the stability of C5-PE38 in mouse serum, C5-PE38 (10 μg/mL) was incubated with mouse serum at 37°C for up to 10 days. To confirm the in vitro integrity and stability of C5-PE38, samples were analyzed by western blotting using an HRP-conjugated anti-His-tag secondary antibody (Sigma, Merck, Germany). In addition, binding functionality of C5-PE38 after 10 days of incubation in mouse serum was assessed using a cell-based ELISA with CLBL-1 cells, as described above.

### 2.5 Immunofluoresence

1.5 × 10^5^ CLBL-1 or 2 × 10^5^ canine PBMCs (control) were plated on a ibidi *μ*-Slide 8 Well Glass Bottom (Ibidi, United States) coated with poly-D-lysine (Gibco, Thermo Fisher Scientific, United States) and incubated for 24 h at 37° C in a humidified atmosphere of 5% CO_2_. Then, 3 μM of C5-PE38 was added to the cells and incubated for 90 min at 37° C. After, cells were fixed with PFA 4% for 15 min, permeabilized with 0.1% Triton X-100 in PBS for 10 min and incubated for 1 h with anti-HA tag Alexa Fluor-488, (1/500 dilution, Invitrogen, Thermo Fisher Scientific, United States). Following incubation, the cells were washed, and ibidi Mounting Medium with DAPI (Ibidi, United States) was added to the wells. Image acquisition was performed on a confocal point-scanning Zeiss LSM 900 with Airyscan 2 microscope (Carl Zeiss, Germany) equipped with a Plan-Apochromat DIC X63 oil objective (1.40 numerical aperture). A diode 405 laser was used to excite DAPI, while Alexa Fluor-488 was excited with the 488 nm blue laser. In the Airyscan acquisition mode, ×1.50 zoom images were recorded at 1024 × 1,024 resolution. ZEN software was used for image acquisition and Fiji software was used for image processing.

### 2.6 Cytotoxicity assay

To determine the cytotoxic activity of C5-PE38 in CLBL-1 and canine PBMC cells, a cell viability assay was performed using the Cell Proliferation Reagent WST-1 (Sigma, Merck, Germany). Briefly, cells were seeded at a density of 6 × 10^4^ per well in 200 μL of culture medium and subjected to increasing concentration (0.15 to 200 μg/mL) of C5-PE38. After 72 h of treatment, cell viability was assessed using WST-1, following the manufacturer’s instructions. Absorbance was measured at 450 nm using an iMark microplate reader (Bio-Rad Laboratories, United States). C5 alone and an irrelevant sdAb fused with PE38 (C1-PE38) were used as controls. Two replicate wells were used to determine each data point and three independent experiments were performed on different days. The best-fit half-maximal effective concentration (EC50) values of each formulation were calculated using GraphPad Prism software (version 9.2.0, San Diego, CA, United States) using the log (inhibitor) *vs* response (variable slope) function.

### 2.7 Evaluation of protein synthesis

To evaluate C5-PE38 mechanism of action the Protein Synthesis Assay Kit (Green; Abcam, United Kingdom) was used according to the manufacturer’s instructions. The protocol was adjusted according to the analysis method: flow cytometry and microscopy. Briefly, for the flow cytometry protocol, 6 × 10^4^ of CLBL-1 cells were seeded into 96-wells plates, resuspended in 100 μL of complete RPMI-1640. Then, increasing concentrations of C5-PE38 (4.5 to 75 μg/mL) were added to the respective wells and the plates were incubated at 37°C in a humidified atmosphere containing 5% CO_2_ for 24 h. The following day, cells were transferred to centrifuge tubes and resuspended in a final volume of 100 μL of the respective compound dilution. Cycloheximide, prepared at a 1:100 dilution from the stock solution according to the kit protocol, was added to the corresponding positive control wells and incubated for 45 min at 37°C. Then, the protein label (2×) was added to all the reactions, except for the negative controls, and incubated for 2 h at 37°C. After incubation, cells were washed with PBS, fixed with fixative solution for 15 min at RT, permeabilized with permeabilization buffer for 10 min at RT, and then, the reaction cocktail (93 μL of PBS, 1 μL of Cooper reagent, 1 μL of fluorescent azide and 5 μL of reducing agent) was added and incubated for 30 min at RT. A final wash with wash buffer was performed and the cells were resuspended in 100 μL of PBS. Finally, the reactions were analyzed using an Attune NxT flow cytometer (Thermo Fisher Scientific, United States). For the microscopy assay, 96 wells-plate were coated with 100 μL of 0.01% poly-L-Lysine, for 1 h and washed with PBS. Then, 6 × 10^4^ of CLBL-1 cells were resuspended in complete RPMI 1640, added to each well, and incubated overnight at 37° C in a humidified atmosphere containing 5% CO_2_. The next day, the medium was removed, and C5-PE38 dilutions were added to the respective wells and incubated for 24 h at 37° C. Cycloheximide controls, fixation, permeabilization and reaction steps were performed as described above. Following this, cells were incubated with DNA Stain (1:1000 dilution) for 20 min. After incubation, the DNA stain was carefully removed, and 100 μL of PBS was added to each well to preserve the samples for imaging. Automated image acquisition was performed on a Leica DMI4000 widefield fluorescence microscope, coupled to Leica DFC365FX 12-bit camera, using Plan-Apochromat x10, x20 or x40 objectives and filtercubes for DAPI and green fluorescence signal, respectively. Basic histogram manipulations were done for illustration purposes in ImageJ/Fiji.

### 2.8 *In vivo* efficacy and biodistribution studies in xenograft model

The *in vivo* efficacy and biodistribution of C5-PE38 were evaluated in a xenograft model of CLBL-1 canine B-cell lymphoma, as described previously (35, 36). All procedures were approved by the Animal Care and Ethical Committee of the Veterinary Medicine Faculty (Protocol_0050132016) and followed the EU recommendations for good practices and animal welfare. Female 6-8-week-old SCID (CB17/Icr-Prkdcscid/IcrIcoCrl) mice were kept in microisolation cages, individually ventilated under pathogen-free conditions, and allowed to acclimatize for 2 weeks. Briefly, a suspension of 1 × 10^6^ CLBL-1 cells in PBS with matrigel was subcutaneously injected into the mice dorsal interscapular region to induce tumor formation. For the efficacy studies when the tumors reached a minimum volume of 100 mm^3^, mice were randomly divided into three groups: control (*n* = 5), C5-PE38 at 0.5 mg/kg (*n* = 5) and C5-PE38 at 1.5 mg/kg (*n* = 5). Treatments were intravenously injected on three consecutive days. Tumor volume was calculated using the formula (width)^2^ × length. C5-PE38 efficacy was determined by tumor growth inhibition (TGI), which was determined as the percentage change in tumor volume of treated over control animals (%T/C). For biodistribution studies, C5-PE38 and C5 (control) were radiolabeled with the radioactive precursor [^99m^Tc(CO)_3_(H_2_O)_3_]^+^ prepared with an IsoLink® kit (Covidien, Ireland). Radiochemical purity was assessed by reversed-phase high-performance liquid chromatography (RP-HPLC) and instant thin-layer chromatography silica gel (ITLC-SG, Agilent Technologies, Santa Clara, CA, United States), as previously described (23). Next, a 10 K Amicon (Merck, Germany) was used for purification and concentration of the ^99m^Tc-labeled C5-PE38 and C5. Then, ^99m^Tc-C5-PE38 or ^99m^Tc-C5 were diluted in PBS and intravenously injected (100 μL) into the tail vein of SCID mice. At 15 min and 3 h post-injection, a group of mice (*n* = 3) were sacrificed by cervical dislocation and radioactivity measured using a dose calibrator (Carpintec CRC-15 W, Ramsey, United States). Tumor and tissues of interest were removed, rinsed, weighted and radioactivity measured using a *γ*-counter (Hidex AMG, Finland). Uptake was represented as a percentage of the injected radioactivity dose per gram of organ or tissue (%ID/g). To confirm the results and C5-PE38 tumor uptake and integrity, western blot analysis was performed as described previously (23). Briefly, tumors, liver, and kidney samples were collected, homogenized, and subjected to immunoprecipitation using His-tag beads (Thermo Fisher Scientific, United States). Immunoprecipitated samples were resolved in a 15% SDS-PAGE acrylamide gel, transferred to membranes, and blocked. The membranes were incubated with HRP-conjugated anti-His antibody (1:3000). Protein detection was performed by chemiluminescence using Luminata Forte Western HRP and visualized on a ChemiDoc XRS+ imaging system (Bio-Rad).

### 2.9 Histopathological and immunohistochemical analysis

Tumor samples were fixed in 10% buffered formalin and embedded in paraffin using a Leica tissue processor. Paraffin blocks were sectioned to obtain 3 μm thick slices, which were mounted on Superfrost Ultra Plus glass slides (Menzel-Gläser, Braunschweig, DE). Hematoxylin and eosin (H&E) staining was performed on selected sections for routine histopathological evaluation. For immunohistochemistry, representative tumor sections were selected, deparaffinized using xylene, and rehydrated using a series of graded ethanol solutions. Antigen retrieval was carried out via microwave treatment in Tris-EDTA buffer (pH 9.0) for 5 min at 900 watts, followed by 15 min at 650 watts. Endogenous peroxidase activity and non-specific binding were blocked using the Peroxidase Block Solution and Protein Block Solution, applied sequentially. The sections were incubated at room temperature for 30 min with polyclonal rabbit anti-human CD20 (1/200, Thermo Fisher Scientific). Nuclear counterstaining was performed with Gill’s hematoxylin for 30 s. Dog lymph node sections were used as positive controls, while sections treated without the primary antibody served as negative controls.

## 3 Results

### 3.1 Construction and expression of C5-PE38

**Figure 1:**
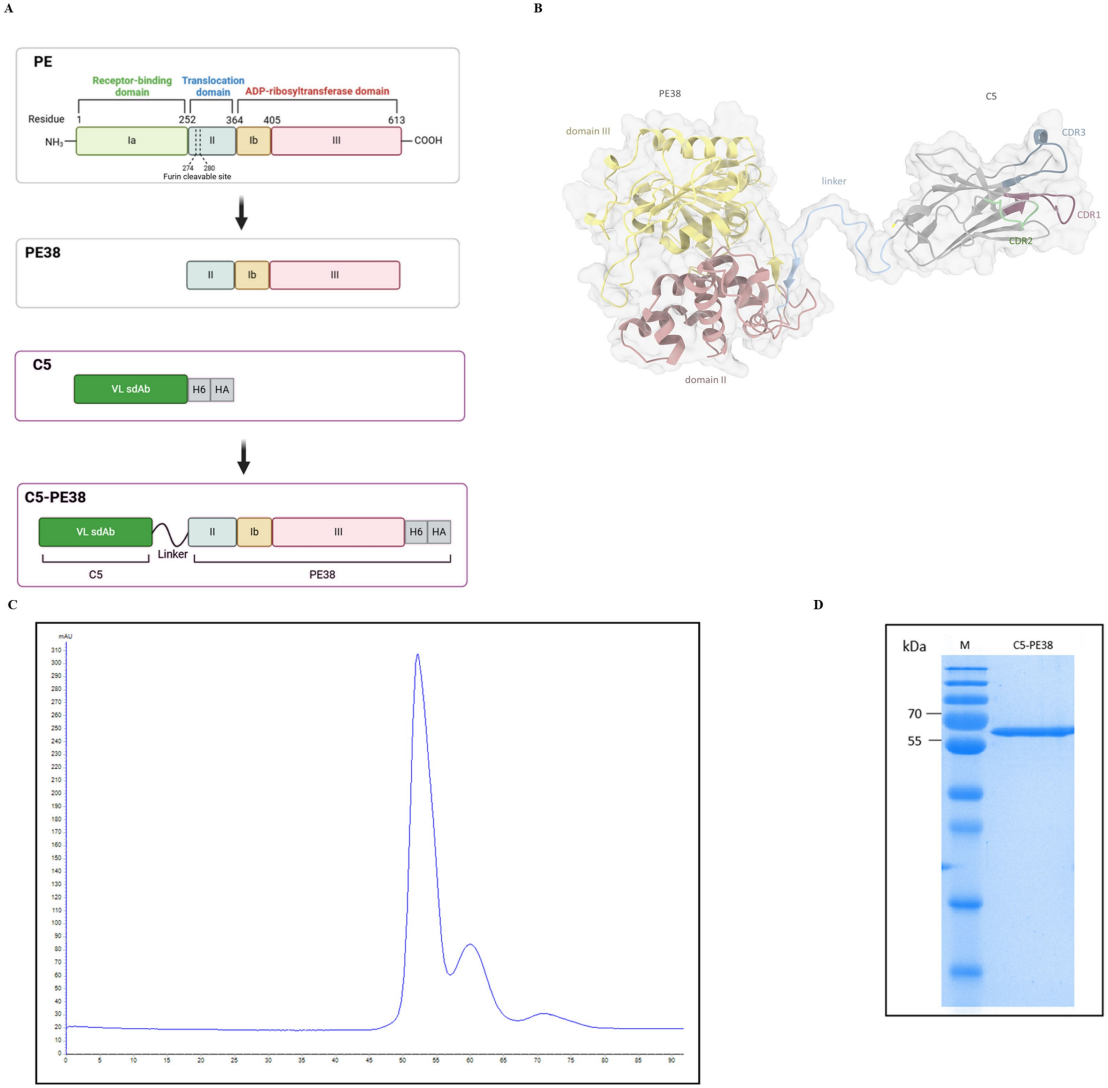
C5-PE38 construction, expression and purification. **(A)** Schematic representation of the construction of the immunotoxin C5-PE38, showing the sequential reduction from full-length Pseudomonas exotoxin A (PE) to the truncated PE38, and the conjugation with the rabbit-derived VL sdAb C5 through a peptide linker (SSGGGGSGGGGGGSSRSS), resulting in the final immunotoxin C5-PE38. The C-terminal His-tag and HA-tag were included in the construct to facilitate purification and detection. **(B)** Ribbon representation of the 3D structure predicted for C5-PE38. The CDR domains of the C5 sdAb are highlighted in purple (CDR1), green (CDR2), and blue (CDR3). Domains II and III of the truncated PE38 are colored red and yellow, respectively. The peptide linker is colored blue. The van der Waals surface is depicted in transparent coloring. The His-tag and HA-tag are not shown in the structural prediction. CDR numbering was performed according to Kabat et al. **(C)** Chromatogram from size exclusion chromatography (SEC) purification of C5-PE38. C5-PE38 was produced using the inclusion bodies protocol (23). The immunotoxin was prepared from a 2-liter culture of *E. coli*, purified using HP HisTrap and SEC, with refolding performed by stepwise dialysis (23). **(D)** The pooled and concentrated C5-PE38 was evaluated by 15% SDS-PAGE following SEC purification. The SDS-PAGE gel confirmed the molecular weight of approximately 58 kDa, as well as the purity and integrity of the immunotoxin. M – Thermo Scientific™ PageRuler™ Plus Prestained Protein Ladder, 10 to 250 kDa.

The recombinant C5-PE38 immunotoxin was generated by molecular biology tools fusing the C5 sdAb with PE38, the truncated portion of *Pseudomonas aeruginosa* exotoxin A, that lacks domain Ia (Figure 1A). C5 is a rabbit-derived sdAb in the VL format that we have recently developed and characterized against canine B-cell lymphoma (23). PE38 is composed of two domains: domain II that contains a furin cleavage site and is essential for toxin activation and domain III that contains ADP ribosylation activity, essential to kill cells (Figure 1A). The C5 sdAb and the PE38 were linked by a flexible linker (SSGGGGSGGGGGGSSRSS). The ribbon representation of the predicted 3D structure for the generated recombinant C5-PE38 is presented in Figure 1B. C5-PE38 was inserted into the pET-21a vector and then was transformed into *Escherichia coli* (BL21) and expressed and purified as described in the material and methods section. C5-PE38 was successfully expressed and purified by HP HisTrap and size exclusion chromatography (SEC; Figure 1C). As shown in Figure 1D, the expected molecular weight of approximately 58 kDa was observed in the 15% SDS-PAGE gel analysis after SEC purification. The final protein yield of C5-PE38 after purification was 5.0 ± 0.2 mg/L.

### 3.2 Binding activity, internalization and stability of C5-PE38

**Figure 2:**
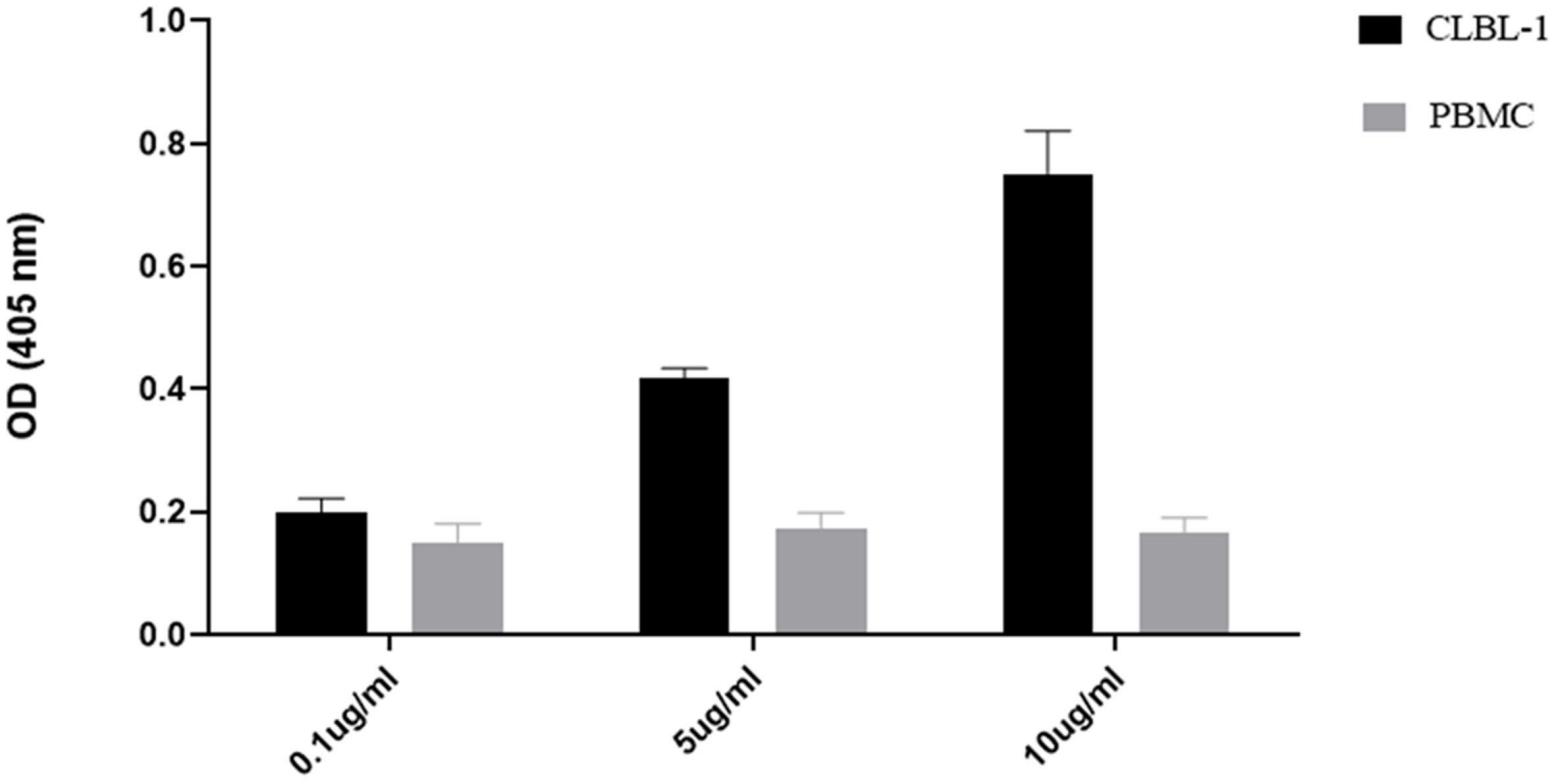
Binding analysis of C5-PE38 against CLBL-1 and canine PBMCs cells. To evaluate the specific binding activity of C5-PE38, a cell-based ELISA was performed against CLBL-1 and PBMCs cells with C5-PE38 at different concentrations (10 *μ*g/mL, 5 μg/mL and 0.1 μg/mL) for 45 min at 4°C. Cells were washed with cold PBS, and anti-HA-HRP was used as secondary antibody and ABTS as substrate solution. The results were measured by optical density at 405 nm and expressed as the mean ± SEM.

**Figure 3:**
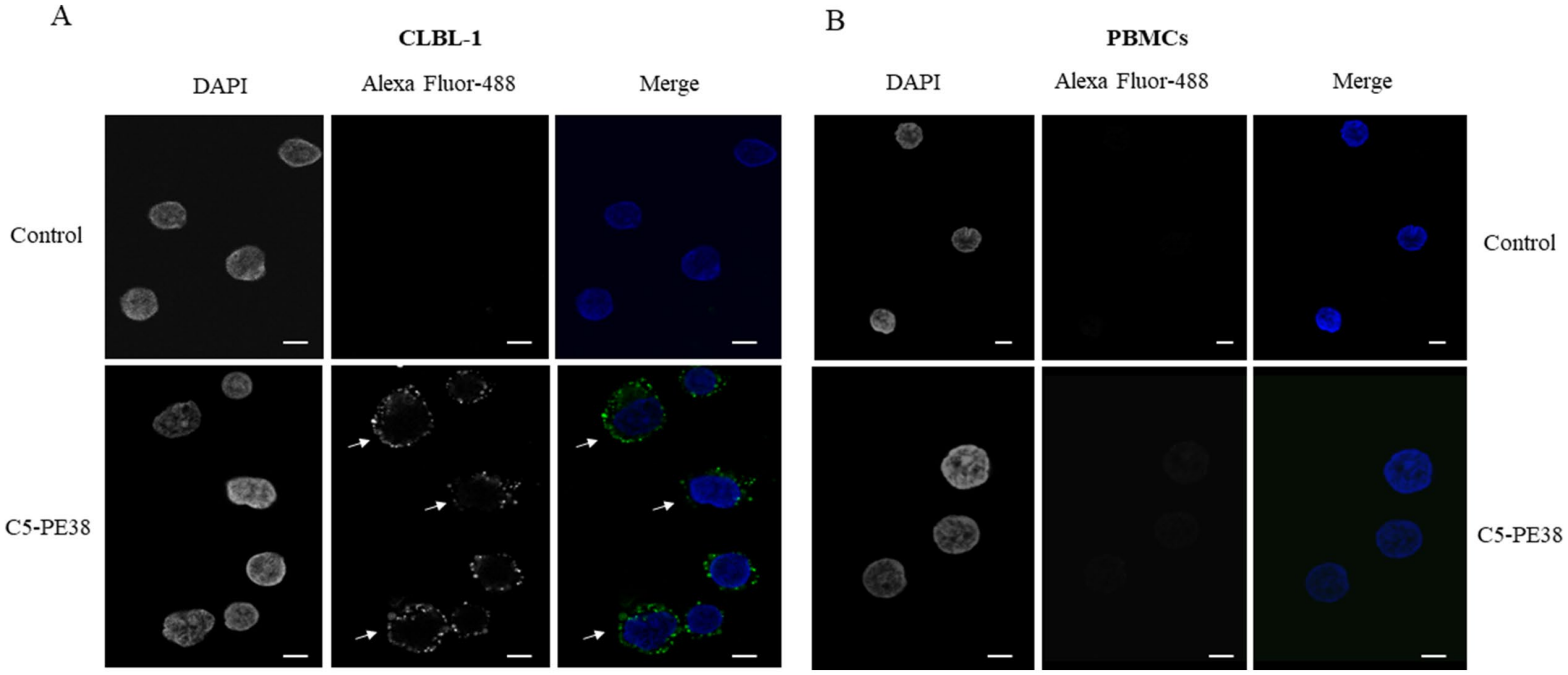
Binding and internalization characterization of C5-PE38. C5-PE38 binding and internalization properties against CLBL-1 and PBMCs were evaluated by immunofluorescence. 1.5 × 10^5^ of CLBL-1 and 2 × 10^5^ PBMCs were plated on ibidi μ-Slide 8 Well Glass Bottom and incubated with 3 μM of C5-PE38 for 90 min. After incubation, cells were washed, fixed, permeabilized, blocked and incubated for 1 h with anti-HA Alexa Fluor-488 (1/500). At the end, ibidi Mounting Medium with DAPI was added to the cells. **(A)** In the CLBL-1 secondary antibody control, no Alexa-488 fluorescence is observed, while in the sample incubated with C5-PE38 it is possible to observe the labeled antibody in the perinuclear region (arrows). **(B)** In contrast, there is no detectable fluorescence in the canine PBMCs. Representative microphotographs with C5-PE38 (green) and DAPI stained nuclei (blue) are shown, scale bar = 5 μm.

**Figure 4:**
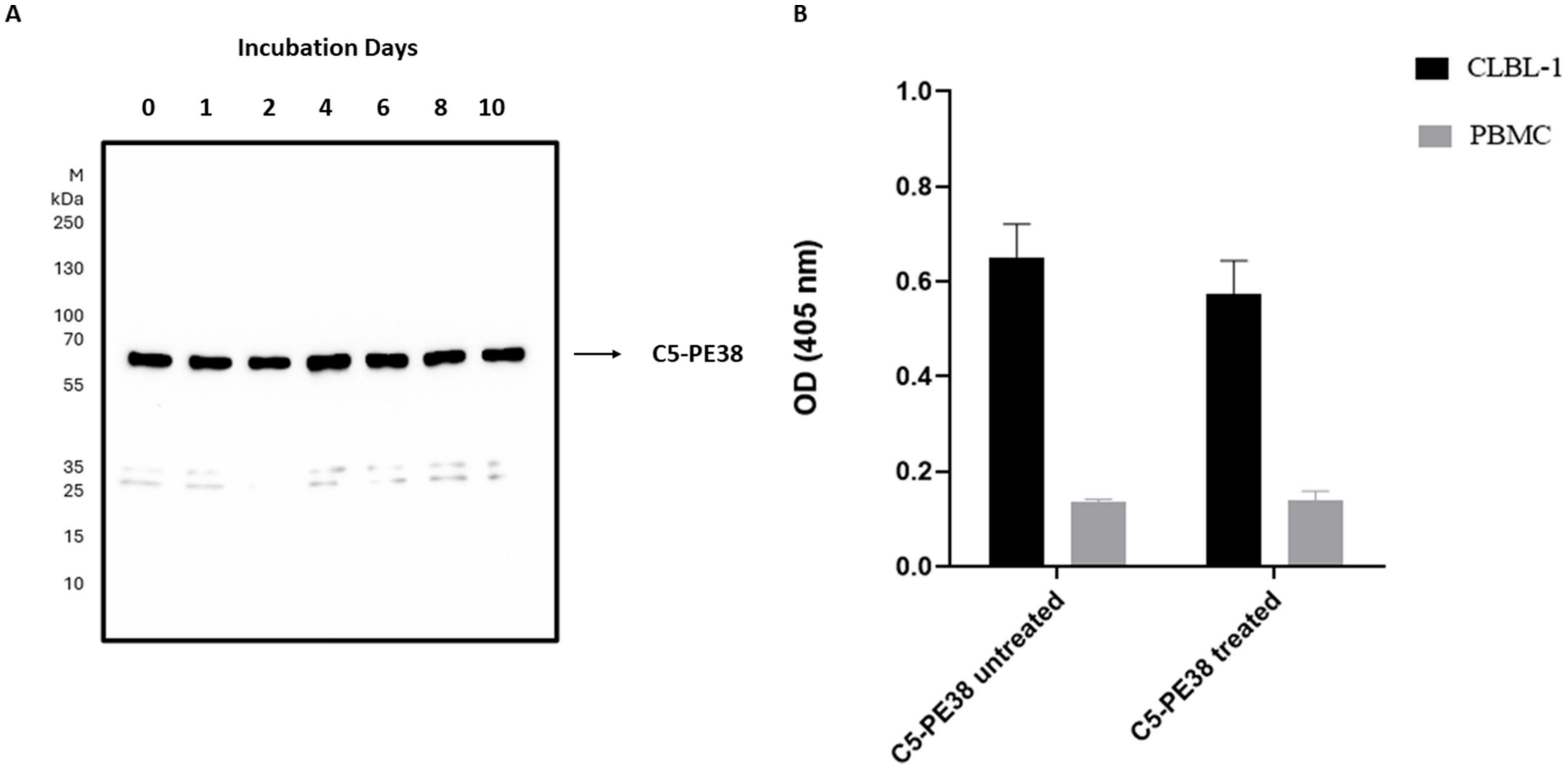
*In vitro* stability and functionality of C5-PE38 in mouse serum. **(A)** The stability of the recombinant C5-PE38 immunotoxin was assessed *in vitro* in mouse serum at 37°C for up to 10 days. Samples were analyzed by Western blotting using an HRP-conjugated anti-His mAb. M: Thermo Scientific™ PageRuler™ Plus Prestained Protein Ladder (10 to 250 kDa). The C5-PE38 immunotoxin, with the expected molecular weight of approximately 58 kDa, was detected at all-time points, showing minimal degradation. **(B)** Binding functionality of C5-PE38 to CLBL-1 cells was evaluated using a cell-based ELISA. Results are shown for the untreated sample and the sample incubated in mouse serum for 10 days at 37°C. The data confirmed that C5-PE38 retained its specific binding capacity, demonstrating stability in both structure and functionality.

To confirm the binding activity and internalization of the recombinant C5-PE38 immunotoxin, cell-based ELISA and immunofluorescence assays were performed. As shown in Figure 2, C5-PE38 exhibited high binding activity toward the CLBL-1 canine B-cell lymphoma cell line. In contrast, no binding activity was observed in the control canine PBMC cells. To better characterize the binding activity of C5-PE38, we evaluated its cellular internalization properties using immunofluorescence. As illustrated in Figures 3A, a prominent accumulation of fluorescence was observed in the perinuclear region of CLBL-1 cells. This fluorescence was detected using an Alexa Fluor-488-conjugated anti-HA antibody, which specifically bound to the HA-tagged C5-PE38. In contrast, no detectable fluorescence above background levels was observed in the control PBMC cells (Figure 3B). These data confirm that C5-PE38 specifically binds to CLBL-1 cells and is internalized into these cells. This binding and internalization feature (most likely into lysosomes) is essential for the development of an effective immunotoxin. To evaluate the *in vitro* C5-PE38 stability, the immunotoxin was incubated with mouse serum at 37°C up to 10 days, and then the integrity was analyzed by western-blot. As shown in Figure 4, C5-PE38 with the expected molecular weight (58 kDa) was detected in all time points and only a slight linker-protein degradation was observed. Moreover, the binding capacity of C5-PE38 to CLBL-1 cells was assessed using a cell-based ELISA, as described above, after 10 days of incubation in mouse serum at 37°C. The results were compared to non-treated protein. Both conditions demonstrated similar binding profiles to CLBL-1 cells, confirming that the immunotoxin retains its binding specificity and functionality after prolonged incubation.

### 3.3 Evaluation of C5-PE38 cytotoxicity

**Figure 5:**
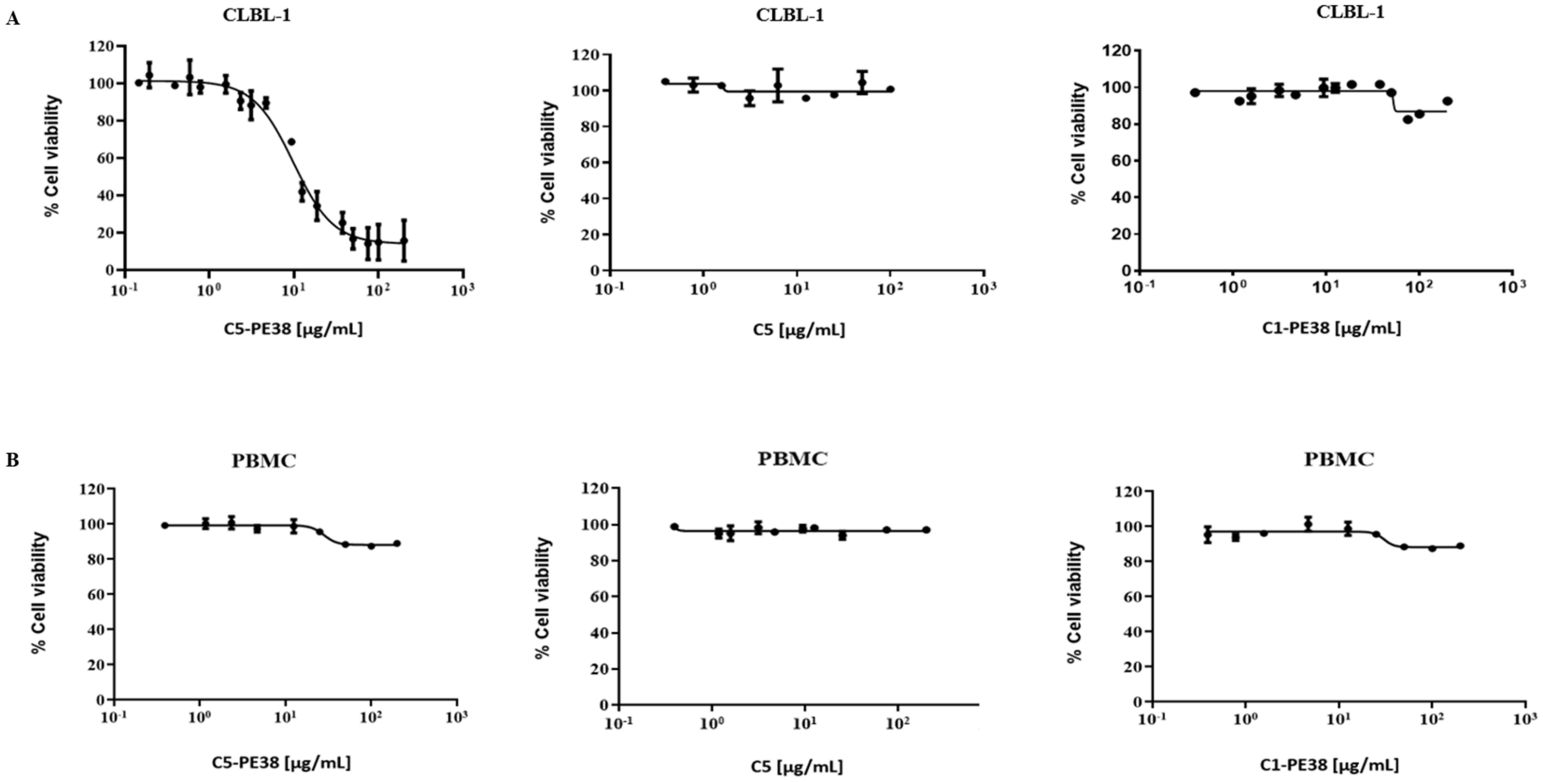
Evaluation of cytotoxic activity of C5-PE38 in CLBL-1 and PBMC cells. To determine the effect of C5-PE38 on CLBL-1 and PBMC, a cell viability assay using the WST-1 reagent was performed. C5 antibody and an irrelevant sdAb (C1) fused with PE38 (C1-PE38) were used as controls. Cells (6 × 10^4^) were seeded and treated with increasing concentrations of each compound (0.15 μg/mL to 200 μg/mL). After 48 h of treatment, the cell viability was assessed using WST-1. **(A)** Evaluation of cytotoxic activity in CLBL-1. C5-PE38 demonstrated a dose-dependent cytotoxic effect on CLBL-1 cell proliferation with an EC50 of 9.50 ± 0.04 μg/mL, demonstrating the specificity of the immunotoxin. In contrast, C1-PE38 and C5 sdAb had no effect on CLBL-1 cells. **(B)** Evaluation of cytotoxic activity in PBMCs. PBMCs were used as a control to evaluate off-target effects, and no significant cytotoxicity was observed, further highlighting the specificity of C5-PE38 for CLBL-1 cells. The best-fit EC50 values of each formulation were calculated using GraphPad Prism software (version 9.2.0, San Diego, CA, United States), using the log (inhibitor) vs. response (variable slope) function.

To evaluate the *in vitro* cytotoxicity of C5-PE38 on canine lymphoma, a cell viability assay was performed on CLBL-1 cells and canine PBMC cells (control) using the WST-1 reagent. As shown in Figure 5A, C5-PE38 exhibited potent cytotoxicity against CLBL-1. Furthermore, dose-dependent cytotoxicity was observed in the CLBL-1 cells in the presence of C5-PE38 (EC_50_ = 9.50 ± 0.04 μg/mL). In contrast, CLBL-1 cell viability was not affected by the presence of C5 alone or an irrelevant sdAb fused with PE38 (C1-PE38), which demonstrates the selective ability of C5-PE38 (Figure 5A). Moreover, C5-PE38, C5 and C1-PE38 had no effect on PBMC cells proliferation (Figure 5B).

### 3.4 Assessment of C5-PE38 activity in inhibition of protein synthesis

**Figure 6:**
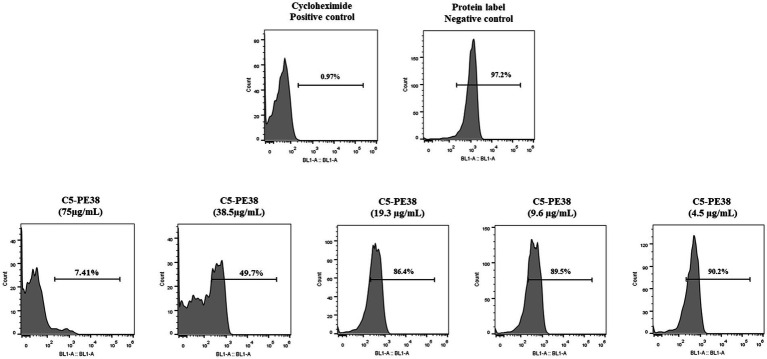
Assessment of protein synthesis inhibition by C5-PE38. To evaluate the mechanism of action of C5-PE38, the Protein Synthesis Assay kit (Abcam) was used with flow cytometry analysis. CLBL-1 cells were treated with varying concentrations of C5-PE38 (4.5 to 75 μg/mL), followed by the manufacturer’s protocol. The data confirm a dose-dependent inhibition of protein synthesis, with higher concentrations (75 μg/mL) effectively inhibiting protein synthesis, while lower concentrations (4.5 μg/mL) had no significant effect, leaving cells viable. Cycloheximide, a known inhibitor of protein synthesis, was used as a positive control to validate the assay. Protein labeling confirmed the synthesis of proteins in untreated cells and their inhibition in treated cells, demonstrating the specific activity of C5-PE38. Additionally, this analysis was complemented by microscopy, which corroborated the dose-dependent inhibition of protein synthesis, as shown in the Supplementary Figure 1.

The main cell death mechanism associated with PE38 toxin is the inhibition of protein synthesis. To confirm that the cytotoxicity induced by C5-PE38 results from the same mechanism, protein synthesis was evaluated by flow cytometry and immunofluorescence using the Protein Synthesis Assay Kit as described in the material and methods section. Cycloheximide, a well-known antifungal agent and inhibitor of protein synthesis, was used as a positive control, while untreated cells served as the negative control. Protein labeling was employed to detect the presence of proteins, in contrast to protein inhibition. The data shown in Figure 6 confirmed that at high concentrations (75 μg/mL), C5-PE38 promoted inhibition of protein synthesis, as low amounts of protein were detected by flow cytometry (Figure 6) and immunofluorescence (Supplementary Figure 1). In contrast, inhibition of protein synthesis of C5-PE38 at lower concentrations was not observed. These results demonstrated a potent and dose-dependent effect of C5-PE38 on the inhibition of protein synthesis.

### 3.5 Antitumor activity and biodistribution of C5-PE38 in a CLBL-1 xenograft model

**Figure 7:**
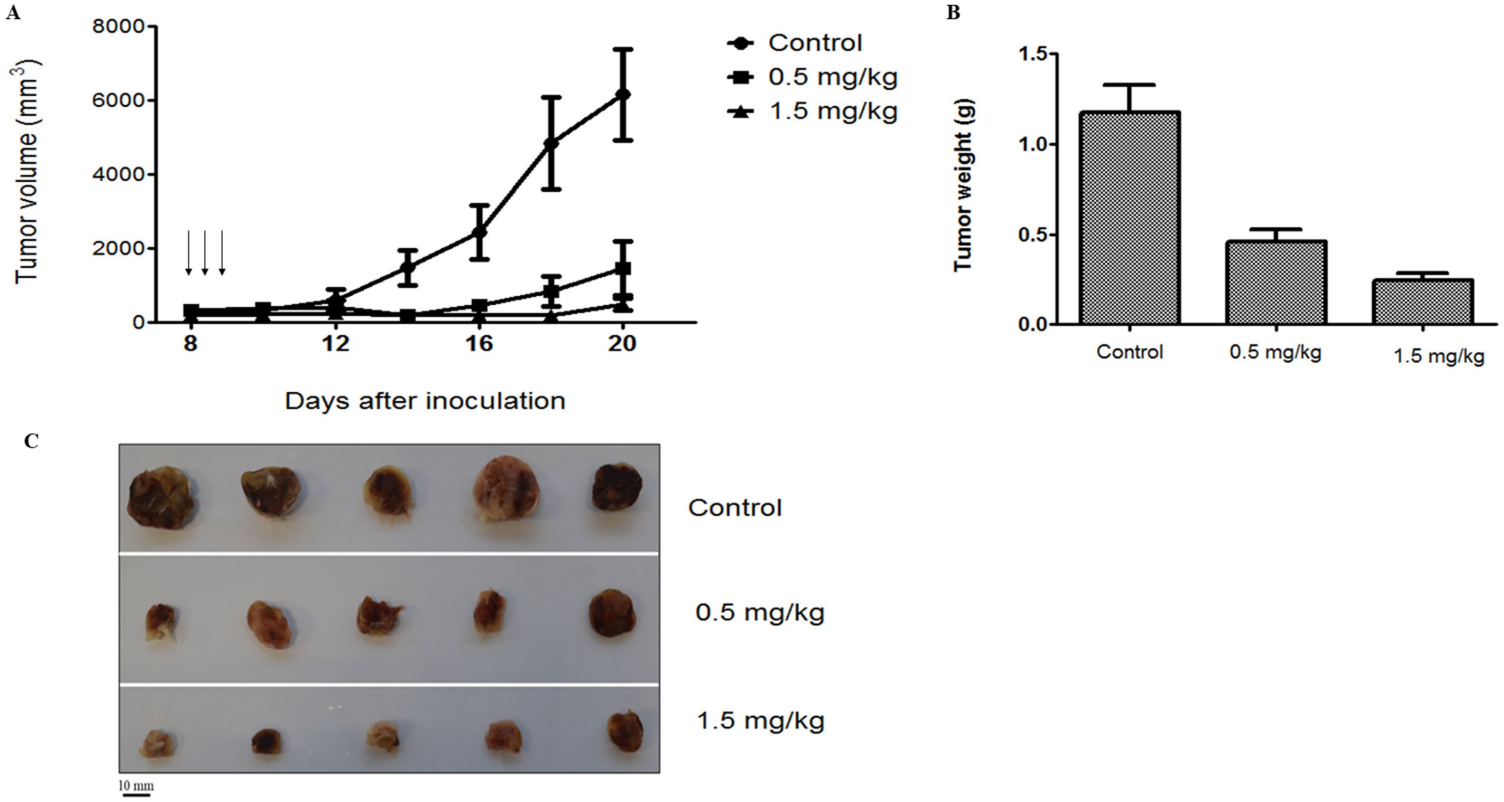
Antitumoral activity of C5-PE38 on CLBL-1 xenograft canine lymphoma model. C5-PE38 Immunotoxin efficacy was *in vivo* evaluated on a xenograft model of canine lymphoma. A suspension of 1 × 10^6^ CLBL-1 cells in PBS with matrigel was subcutaneously injected into the dorsal intrascapular region of female 6-8-week-old SCID (CB17/Icr-Prkdcscid/IcrIcoCrl) to induce tumor formation. When the tumors reached a volume of 100 mm^3^, mice were divided in 3 groups: untreated/control (PBS vehicle only, *n* = 5), C5-PE38 at 0.5 mg/Kg (*n* = 5) and C5-PE38 at 1.5 mg/kg (*n* = 5). Three treatments were intravenously administrated for three consecutive days (arrows). **(A)** Tumor volume was calculated using the formula (width)^2^ x length. Efficacy was determined by tumor growth inhibition (TGI) that is determined as the percentage change in tumor volume of the treated over control animals (%T/C). **(B)** Tumor weight of CLBL-1 xenograft. **(C)** Representative images of xenograft tumors were captured at the end of 2 weeks of treatment. Scale bar = 10 mm.

**Figure 8:**
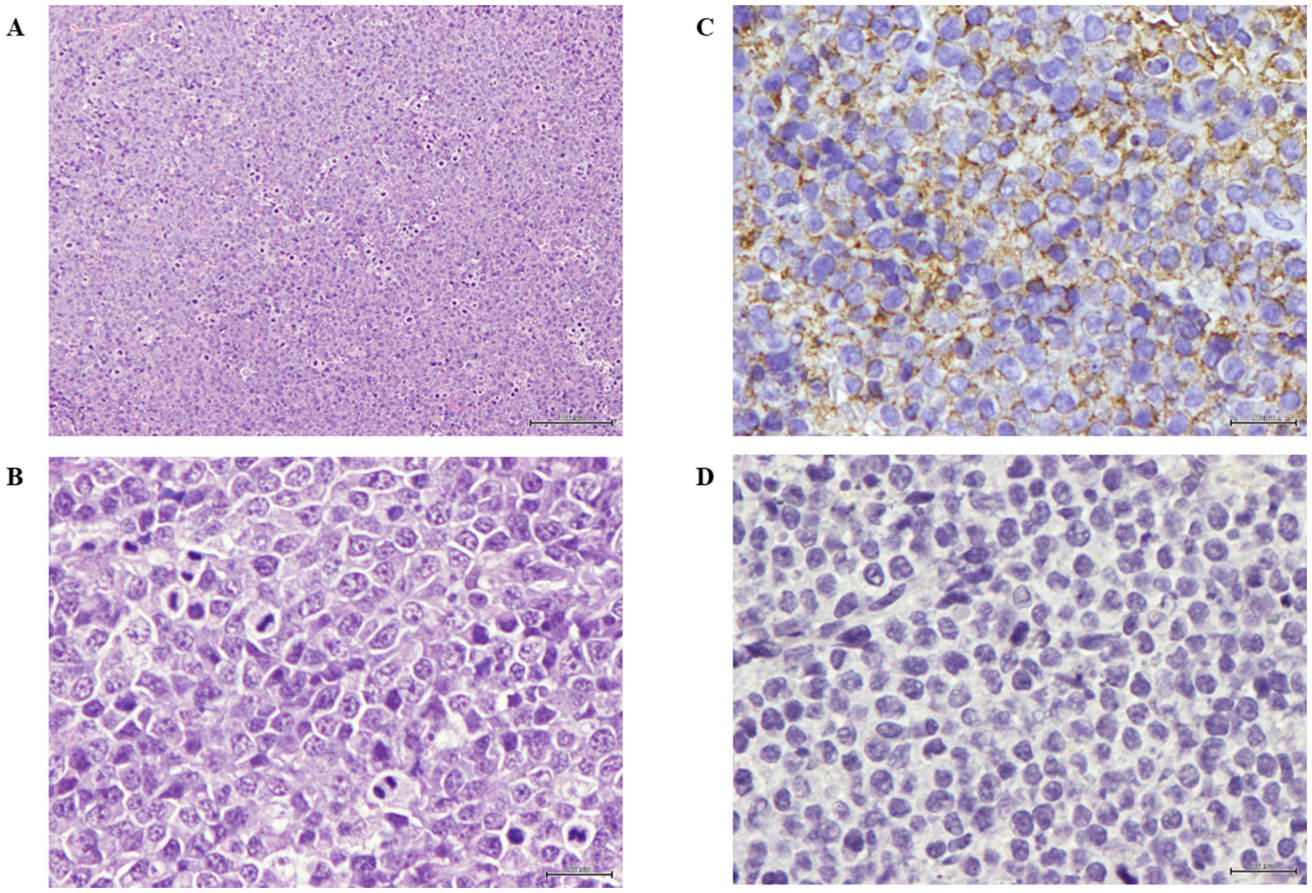
Histopathological and immunohistochemical analysis of C5-PE38. **(A)** Xenograft tumor section presenting neoplasia classified as high-grade centroblastic diffuse malignant lymphoma. The neoplasia is composed of monomorphic large cells with high cellular density and a starry-sky pattern. Stained with hematoxylin and eosin (H&E). Magnification: 100×; scale bar: 100 μm. Hematoxylin and eosin (H&E) stained. Magnification = 100×, scale bar = 100 μm. **(B)** Xenograft tumor section presenting lymphoma composed of monomorphic round cells, with several marginal and small nucleoli per cell and high mitotic index. Hematoxylin and eosin (H&E) stained. Magnification = 400×, scale bar = 20 μm. **(C)** Xenograft tumor section analyzed using immunohistochemistry for B-cells, presenting membranous positivity in virtually 100% of tumor cells. Stained with anti-CD20 antibody and counterstained with Gill’s hematoxylin. Magnification = 400×, scale bar = 20 μm. **(D)** Xenograft tumor section analyzed using the immunohistochemistry technique for T-cells, showing a lack of marker expression in tumor cells. Stained with anti-CD3 antibody and counterstained with Gill’s hematoxylin. Magnification = 400×, scale bar = 20 μm.

The antitumor effect and biodistribution profile of C5-PE38 were evaluated in a murine xenograft model of canine B-cell lymphoma. For this purpose, a CLBL-1 cell suspension (1 × 10^6^ cells) was injected subcutaneously into the dorsal interscapular region of mice severely combined immunodeficient (SCID). When tumors reached a volume of ~100 mm^3^, mice were randomized into three groups: untreated/control (PBS vehicle only, *n* = 5), C5-PE38 at 0.5 mg/kg (*n* = 5), and C5-PE38 at 1.5 mg/kg (*n* = 5). Treatments were intravenously injected for three consecutive days, and tumor volumes and body weights were measured daily for 2 weeks. As shown in Figures 7A–C, tumor growth was halted and began to regress immediately after C5-PE38 treatment at both doses was initiated. The maximum tumor growth inhibition (TGI) effect was strongly evident at both 0.5 mg/kg and 1.5 mg/kg treatment dosages, inhibiting tumor growth by 76.2 and 92.3% respectively, when compared to vehicle control treated mice. These results confirmed the strong antitumor activity of the C5-PE38 in a murine canine B-cell lymphoma xenograft model. Moreover, it is important to mention that histological and immunohistochemical analyses confirmed that the xenograft tumors retained the histological characteristics of the original CLBL-1 cell line. Specifically, the tumors exhibited positive expression of B-cell markers and an absence of T-cell marker expression, consistent with the phenotype of the original cell line xenografts. These findings are presented in Figure 8, further demonstrating that the tumor model faithfully represents the biological properties of canine B-cell lymphoma.

**Figure 9:**
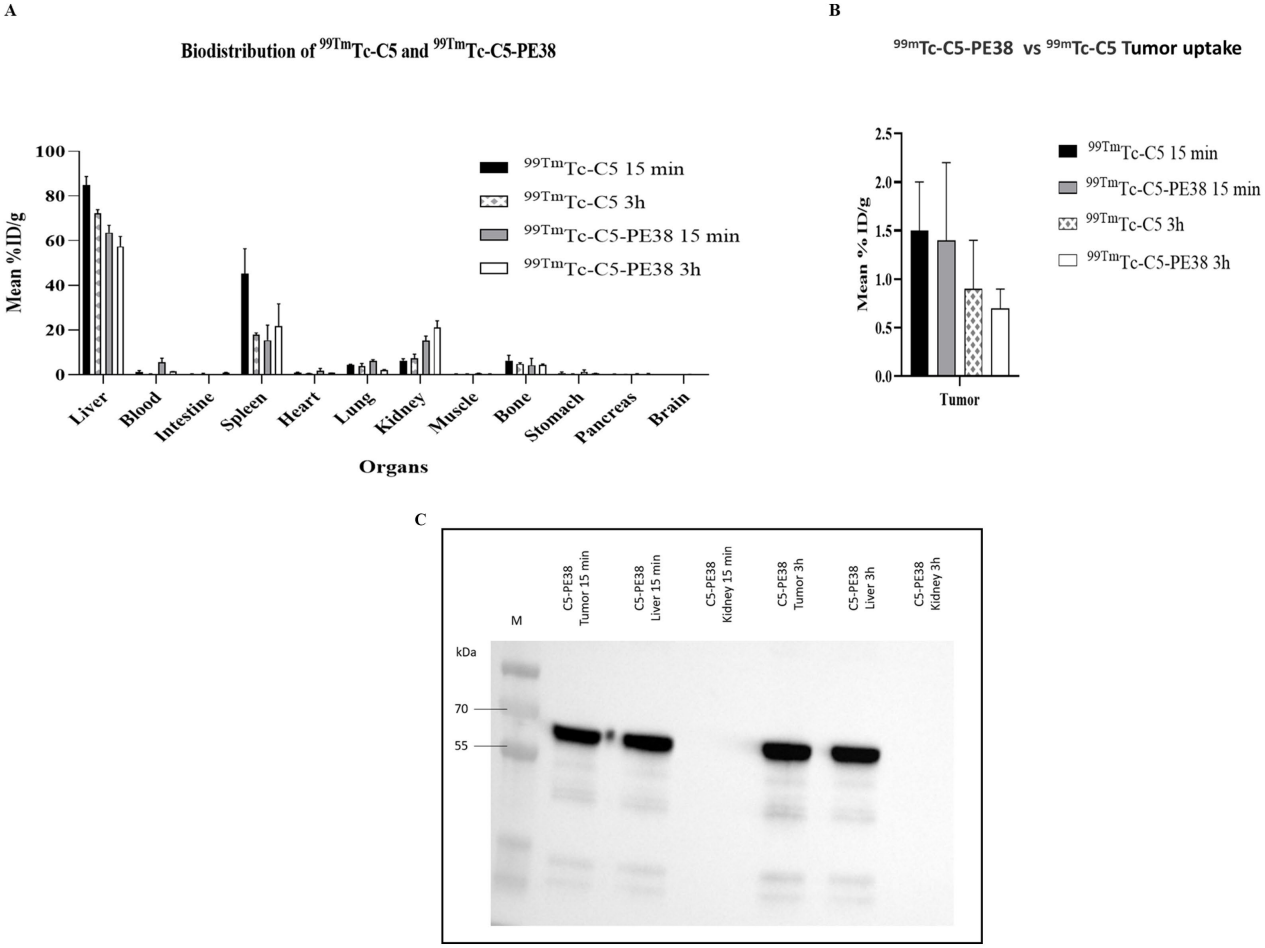
Biodistribution profile of 99mTc-C5-PE38 and 99mTc-C5. To evaluate the tumoral uptake and pharmacokinetic profile of the C5-PE38 immunotoxin and C5 (control), a biodistribution assay was performed on a CLBL-1 xenograft model at two different time points (15 min and 3 h). C5-PE38 and C5 were radiolabeled with 99mTc(CO)3(H2O)3 and intravenously injected in the tail vein of a xenograft mouse model. Mice were sacrificed at 15 min and 3 h, and the radioactivity of each organ was measured. The activity in each organ was calculated and expressed as a percentage of injected radioactivity dose per gram of organ or tissue (%ID/g). **(A,B)** The results show that the tumoral uptake was around 2 ± 0.5%ID/g at 15 min, diminishing to 1 ± 0.5% at 3 h after injection. **(C)** Tumor uptake and the integrity of C5-PE38 were confirmed by Western blot analysis. Tumors, liver, and kidney samples were collected, homogenized, and subjected to immunoprecipitation using His-tag beads. Immunoprecipitated samples were separated on a 15% SDS-PAGE gel, transferred to a membrane, and blocked. The membrane was incubated with an HRP-conjugated anti-His antibody (1:3000), and protein detection was performed using chemiluminescence with Luminata Forte Western HRP. Results were visualized using the ChemiDoc XRS+ imaging system (Bio-Rad). Representative blots confirm the presence of C5-PE38 in CLBL-1 xenograft tumors at 15 min and 3 h post-injection, as well as in the liver, consistent with the biodistribution data.

To evaluate the biodistribution and tumor uptake of C5-PE38 in the CLBL-1 xenograft model, ^99m^Tc-labeled C5-PE38 and C5 (control) molecules were intravenously injected into the tail vein of SCID mice, sacrificed at 15 min and 3 h and then the radioactivity was measured as described in the Materials and Methods section. The data obtained, expressed as the percentage of injected activity per gram of organ (%ID/g), are shown in Figures 9A,B. Analysis of the biodistribution data of ^99m^Tc-C5-PE38 revealed a rapid hepatic and splenic uptake associated with a small urinary tract excretion (15.3 ± 2.0% ID/g and 5.4 ± 0.4% at 15 min in the kidneys and total excreted activity, respectively). Although the hepatobiliary elimination of the ^99m^Tc-C5-PE38 from the bloodstream seems slightly decreased compared to the non-conjugated ^99m^Tc-C5 sdAb, the obtained immunotoxin biodistribution profile appears to be grossly identical. Importantly, tumor uptake was also similar to C5, being around 2.0% ± 0.5 ID/g at 15 min after injection and further decreasing to approximately 1.0% ± 0.5 ID/g after 3 h of injection. Furthermore, tumor uptake and the integrity of C5-PE38 were confirmed by western blot analysis (Figure 9C). The results demonstrated specific targeting and uptake of C5-PE38 in CLBL-1 xenograft tumors at both 15 min and 3 h post-administration. The immunotoxin was detected at its expected molecular size in tumors, confirming its structural integrity *in vivo*. In line with the biodistribution data, C5-PE38 was also detected in the liver, as anticipated, corroborating its hepatic uptake. These findings underscore the targeting specificity, stability, and favorable biodistribution profile of C5-PE38, reinforcing its potential as a therapeutic agent.

## 4 Discussion

The entry of immunotoxins into the market for cancer treatment has created new possibilities for oncologic patients, particularly for those with acquired tumor drug resistance due to treatments with conventional cytotoxic molecules. Immunotoxins induce cell death by disrupting protein synthesis, offering a novel mechanism to treat resistant cancers. This unique mechanism of action allows immunotoxins to be combined with standard agents to target quiescent cells, reducing cross-resistance with conventional treatments. The development of effective immunotoxins requires the identification of targets with highly differential expression between cancer cells and normal tissues to ensure specificity and minimize side effects. Therefore, selecting an appropriate antibody is critical to avoid undesired toxicities (37).

The clinical efficacy of an immunotoxin comprising an antibody fragment was demonstrated in 2018 with the approval of Moxetumomab pasudotox, an Fv antibody fragment fused to PE38, for the treatment of relapsed and refractory human hairy cell leukemia (14, 38). Despite the promising potential of immunotoxins, of the three approved immunotoxins, Moxetumomab pasudotox remains the only antibody-derived immunotoxin approved for human use, and no immunotoxins have been approved for veterinary applications. This highlights both the emerging stage of regulatory acceptance and the potential for future developments of immunotoxins in human and veterinary medicine.

In this context, our study aimed to explore the promising properties of rabbit-derived sdAbs to develop a new generation of recombinant immunotoxins for canine B-cell lymphoma treatment, while contributing to comparative oncology. For this purpose, we fused the truncated PE38 toxin domain derived from *Pseudomonas aeruginosa* exotoxin A with the rabbit-derived sdAb C5 clone, which we recently developed and characterized for canine B-cell lymphoma (23). To construct our immunotoxin, we explored the potent, well-characterized and clinically validated PE toxin. PE is a potent toxin produced by *Pseudomonas aeruginosa* (39). Although its mechanism of action is complex and not fully understood, it is well known that PE catalyzes the transfer of ADP ribose from nicotinamide adenine dinucleotide to the elongation factor 2 (EF2) (40). This modification irreversibly inactivates EF2, leading to protein translation arrest and apoptosis. Recombinant immunotoxins are generated by replacing the binding domain of the toxin with the antibody binding fragment portion of an antibody that directs the toxin to cancer cells. Various studies have reported the use of sdAbs and nanobodies (VHHs), which are naturally derived single-domain antibodies from camels and llamas, as moieties for targeted delivery of the PE bacterial toxin. PE and its derived domains have been fused to anti-GPC3 (41, 42), anti-GPC2 (43), anti-VEGFR2 (44), anti-CD7 (45, 46), anti-HER2 (47), and anti-CD38 (48) nanobodies to improve their cytotoxic activities in several tumor models. In the present study, we developed a recombinant immunotoxin using the C5 sdAb, a promising rabbit-derived sdAb antibody targeting canine B-cell lymphoma, previously developed by our group. The C5 sdAb was selected using an *in vivo* phage display selection approach in a xenograft mouse model of canine B-cell lymphoma. This sdAb has been characterized and validated as a suitable scaffold for the construction of an antibody-drug conjugate (ADC) by fusing the C5 sdAb with the SN-38 cytotoxic compound (23).

Herein, molecular biology methods were used to fuse the C5 sdAb with PE38 toxin to construct the recombinant immunotoxin. Instead of conventional chemical conjugation, genetic methods have been introduced in the 3rd generation of immunotoxin, revolutionizing the development of new immunotoxins. The evolution of molecular biology methods easily allowed the fusion of antibody fragments to truncated toxins via a peptide linker. Our data demonstrated that the PE38 toxin could be efficiently fused to the sdAb and expressed in *E. coli* with a high protein yield recovery per liter. Moreover, the results obtained demonstrated that C5-PE38 exhibits high specificity and binding activity toward the CLBL-1 canine B-cell lymphoma cell line, as shown by cell-based ELISA and immunofluorescence assays. The immunotoxin was effectively internalized into the cytoplasm of CLBL-1 cells, a crucial feature for its cytotoxic action. Stability studies confirmed that C5-PE38 maintains its integrity and binding activity when incubated in mouse serum at 37°C for up to 10 days. This stability is essential for its potential therapeutic use, ensuring that the immunotoxin remains effective under physiological conditions.

The cytotoxicity assays showed that C5-PE38 presented a strong cytotoxic effect in CLBL-1 canine B-cell lymphoma cells, which was further linked to the inhibition of protein synthesis. The antitumor activity of C5-PE38 was further tested *in vivo* using a CLBL-1 murine xenograft model. The data obtained confirmed the strong antitumor activity of the immunotoxin at both 0.5 mg/kg and 1.5 mg/kg treatment doses. To evaluate tumor uptake and reinforce the specificity of the immunotoxin toward canine B-cell lymphoma, biodistribution studies were performed using ^99m^Tc-labeled C5-PE38. Fast elimination from the main organs was verified; however, C5-PE38 accumulation was observed in the liver, kidney, and spleen, which could be related to excretory pathways. Tumor uptake data showed a time-dependent decrease, highlighting the rapid uptake of C5-PE38. The obtained biodistribution profile closely resembles that of C5 sdAb, revealing that conjugation with PE38 preserves the specificity of C5 sdAb. These findings highlight the significant potential of C5-PE38 to specifically eliminate tumor cells to treat canine B-cell lymphoma. However, to advance our immunotoxin toward clinical applications, it is essential to address several key challenges. A critical step involves “caninizing” the sdAb to minimize immune responses in dogs. Additionally, incorporating a de-immunized PE38 domain (49) could significantly lower the possible adverse immune reactions that might occur against PE38, thereby improving the safety profile of the immunotoxin. Another challenge is to improve the half-life of the immunotoxin, since a short half-life can limit therapeutic efficacy by requiring frequent dosing. We recently demonstrated a proof-of-concept for a promising albumin domain derived from *Streptococcus zooepidemicus* as a novel strategy for improving the pharmacokinetic properties of therapeutic molecules (50). We aim to incorporate this domain to enhance the half-life of our immunotoxin. Addressing these issues will be crucial for making our immunotoxin a viable therapeutic option in clinical settings. Moreover, efforts are underway to further characterize the C5-PE38 molecule through immunoprecipitation, mass spectrometry studies, and advanced immunofluorescence assays to identify its specific binding target and elucidate its intracellular behavior and properties. Parallel efforts are also focused on the C5 caninization and PE38 de-immunization to reduce the immunogenicity of the recombinant C5-PE38 immunotoxin. Together, these studies will provide valuable insights into the characterization of the immunotoxin and support the further optimization of its design, ultimately enhancing its clinical potential.

Overall, the results obtained herein validated the PE38 toxin as a promising therapeutic molecule for the treatment of dog-related tumors, and rabbit-derived sdAbs as a suitable scaffold for the development of immunotoxins. Some studies have already used dogs as models to evaluate immunotoxins. For instance, Henry *et al.* used canine models of spontaneous invasive carcinomas to evaluate the efficacy of the BR96 sFv-PE40 immunotoxin and achieved successful results in treating certain carcinomas (51). Similarly, Damle *et al.* studied the immunogenicity of the BMS-191352 immunotoxin in dogs (52). Also, dogs have been used to evaluate a novel EGF-anthrax toxin chimera developed for the treatment of bladder cancer (53). To the best of our knowledge, the study described herein is the first attempt to develop an immunotoxin for canine B-cell lymphoma, as determined through a comprehensive search of the literature using PubMed. The development of C5-PE38 represents a significant step forward in the development of targeted therapies for canine B-cell lymphoma. Its high specificity, stability, potent cytotoxicity, and strong antitumor activity underscores its potential as a novel therapeutic option. Moreover, our study contributes to the field of comparative oncology by highlighting the potential use of canine models to develop and evaluate novel cancer therapeutics. Dogs with spontaneous tumors, such as lymphoma, offer valuable insights into the effectiveness and safety of new treatments that could also be relevant to human cancers. Our approach can be translated to other cancer types, contributing to comparative oncology models and advancing some of the challenges associated with immunotoxins. In conclusion, this study opens new perspectives for the use of immunotoxins as a promising treatment for canine B-cell lymphoma, as well as reinforcing dogs as an animal model for the development of new therapeutic molecules against non-Hodgkin lymphoma and other cancer types.

## Data Availability

The original contributions presented in the study are included in the article/supplementary material, further inquiries can be directed to the corresponding author.
