# Supplementary figures and images for: A new treatment for canine B-cell lymphoma based on a recombinant single-domain antibody immunotoxin derived from Pseudomonas aeruginosa exotoxin A
Source: Front Vet Sci. 2025 Apr 4;12:1491934. doi: 10.3389/fvets.2025.1491934 (PMC12007482; doi:10.3389/fvets.2025.1491934)

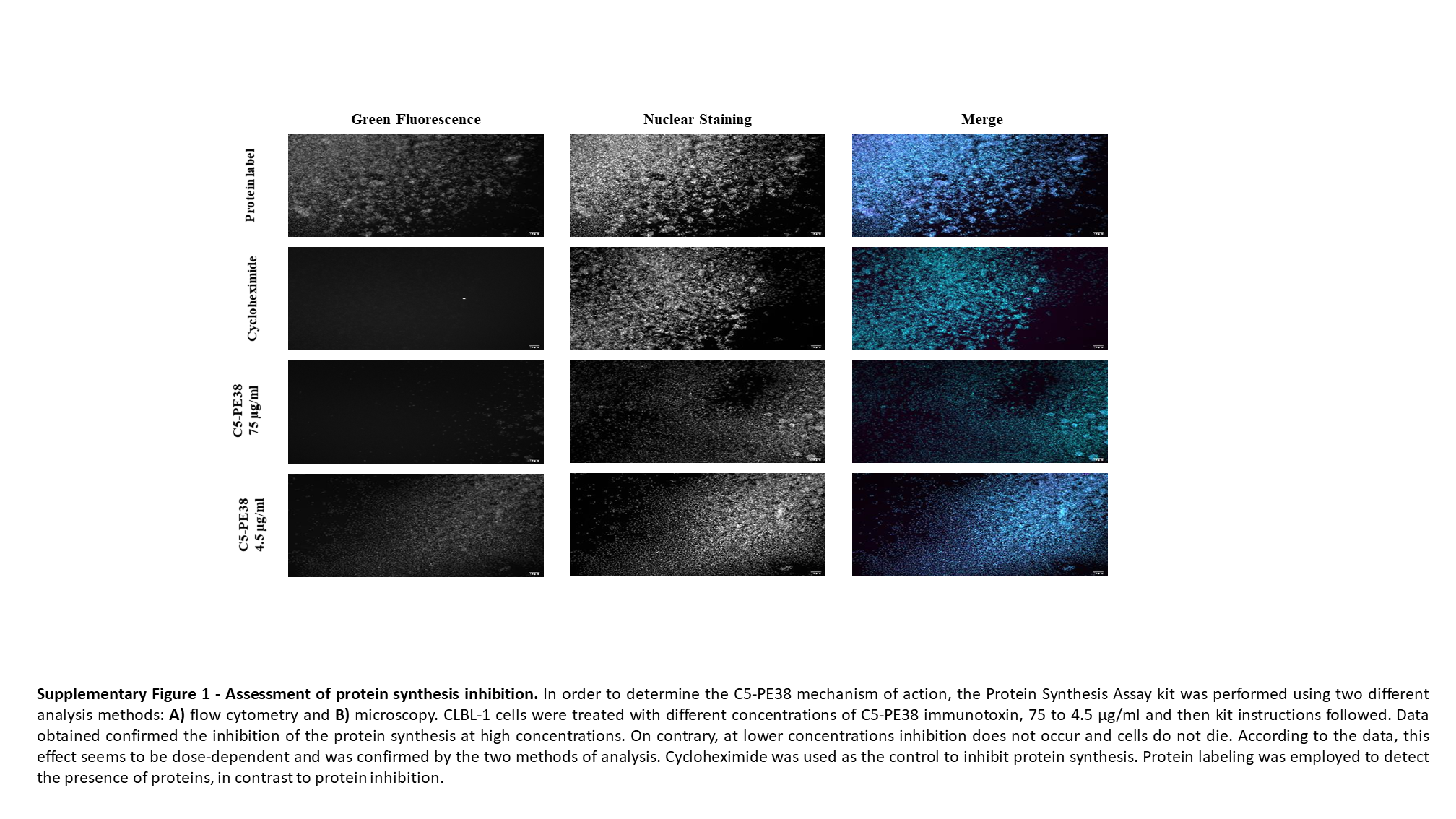

Supplement: Supplementary file 1 [file Image_1.tif]
